# The Prevalence and Risk Factors of Gastric Polyp in Asymptomatic Patients Receiving Health Examination

**DOI:** 10.1155/2018/9451905

**Published:** 2018-12-10

**Authors:** Fu-Wei Wang, Su-Chi Young, Ru-Yih Chen, Kung-Hung Lin, Yan-Hua Chen, Ping-I Hsu, Hsien-Chung Yu

**Affiliations:** ^1^Department of Family Medicine, Kaohsiung Veterans General Hospital, Kaohsiung City 81362, Taiwan; ^2^Department of Nursing, Meiho University, Pingtung City 912, Taiwan; ^3^Center for Geriatrics and Gerontology, Kaohsiung Veterans General Hospital, Kaohsiung City 81362, Taiwan; ^4^Division of Gastroenterology, Department of Internal Medicine, Kaohsiung Veterans General Hospital, Kaohsiung City 81362, Taiwan

## Abstract

**Background:**

To determine the risk factors and prevalence of asymptomatic gastric polyps in the general population of Taiwan.

**Methods:**

Consecutive asymptomatic individuals completing a health examination during October 2015–March 2016 were enrolled in the study and subjected to upper GI endoscopy. Their demographic data and medical history were collected, and the prevalence of gastric polyps was calculated. The risk factors of gastric polyps were identified by analyzing these data through univariate and multivariate logistic regression.

**Results:**

Gastric polyp prevalence in the study population was 29.8%. Age range of 45–60 years, current smoking, and lack of regular exercise were found to be significantly associated with gastric polyps, whereas age range of 45–60 years (relative risk [RR], 1.59; 95% confidence interval [CI], 1.06–2.40) and current smoking (RR, 1.63; 95% CI, 1.04–2.55) were found to be independent predictors for gastric polyps.

**Conclusions:**

In Taiwan, asymptomatic gastric polyps have a prevalence of 29.8%. Age range of 45–60 years and current smoking may increase the risk of asymptomatic gastric polyps.

## 1. Introduction

The rate of diagnosis, mostly incidental, of gastric mucosal lesion is increasing because of the growing adoption of endoscopy in clinical practice. Gastric polyps exhibit varied and diverse pathologies; most cases are asymptomatic, but such symptoms as abdomen pain, anemia, bleeding, and gastric outlet obstruction may accompany large polyps. Some types of polyps can be identified through endoscopy given their typical appearance, but a histology study is essential to detect dysplasia. Hence, all cases of gastric polyps should be appropriately assessed to evaluate the premalignant risk. Polyps may be an indicator of both a genetic disease and a high malignancy risk [1, 2]. Thus, identifying the risk factors of gastric polyps would likely lead to the stratification of the risks as well as to the development of risk amelioration strategies.

Studies have reported that gastric polyps exhibit ethnic, geographic, and other variabilities. For example, approximately 3–5% of patients subjected to upper endoscopy have been found to have benign gastric polyps [3, 4]. With relative prevalence of nearly 70%, hyperplastic polyps are the most prevalent type of polyps [5, 6]. Since the early 2000s, reports especially from Western countries have been revealing sharp increase in the occurrence of fundic gland polyps [4, 7]. Furthermore, a recent nationwide study in the United States examined the occurrence of gastric polyps during 2007-2008 and found the following distributions: fundic gland polyps, 77%; hyperplastic polyps, 17%; and adenoma, 0.69% [4].

The risk factors of gastric polyps are unclear at present, and the underlying pathologies for gastric polyps among Asian population are rather inconsistent [8, 9]. Given the relatively higher background prevalence of Helicobacter pylori infection in Taiwan [10, 11] and because some individuals in Taiwan undertake health examinations, inclusive of upper GI endoscopy (and pay for it themselves), we performed this cross-sectional study to determine the risk factors and prevalence of gastric polyps in Taiwan.

## 2. Methods

### 2.1. Subjects

From October 2015 to March 2016, consecutive asymptomatic individuals of age 20 years or older completing an upper GI endoscopy as part of a health examination were enrolled in the present study. The exclusion criterion was the presence of symptoms of upper gastrointestinal tract disease such as symptoms of reflux and dyspepsia (e.g., dysphagia, epigastric fullness or pain, heartburn, nausea, regurgitation, and vomiting) in the preceding 2 weeks that would typically require medical evaluation. Written informed consent was obtained from all enrolled participants. The study protocol was approved by the Institutional Review Board of Kaohsiung Veterans General Hospital.

### 2.2. Study Design

Each individual completing the health examination was subjected to complete history taking, a physical examination, and a comprehensive review to detect any abdominal symptoms presenting in the preceding 2 weeks. Individuals who satisfied the aforementioned inclusion criteria were categorized as asymptomatic participants and enrolled in the study. All included participants were administered anthropometric tests. In addition, after the participants had fasted overnight, they were examined for gastric mucosa lesions through total upper GI endoscopy (Olympus GIF-Q290 and GIF-H290Z, Olympus Corp., Tokyo, Japan) under the guidance of four experienced endoscopists, namely, Chen YH, Hsu PI, Lin KH, and Yu HC. In this study, any instance of protuberance into the lumen from the gastric mucosa, which is typically flat, was considered a gastric polyp. All visible polyps were either biopsied or removed and thus identified and studied them histologically by the pathologist and classified them as adenomatous polyps, hyperplastic polyps, fundic gland polyps, and other polyps.

The following data of the participants were collected and categorized for analysis: age; body mass index (BMI); consumption statuses of alcohol, cigarettes, betel nut, coffee, spicy food, and tea; consumption status of meat (i.e., vegetarian diet or not); educational level; exercise habits; family history of gastric cancer; gender; long-term use of nonsteroidal anti-inflammatory drug (NSAID); waist circumference; and Helicobacter pylori infection.

### 2.3. Statistical Analysis

The associations between the aforementioned clinical characteristics and the development of gastric polyps were assessed through either the chi-square test or Fisher's exact test. These following variables were included in the analysis (categorization shown within parentheses): age (<39, 40–49, 50–59, 60–69, and >70 years); betel nut habit (yes and no); BMI (<25, 25–30, and >30); consumption of alcohol, coffee, tea, or spicy food (0, ≤ 3, and >3 times per week); education level (<10, 10–12, and >12 years); exercise habit (0, ≤ 3, and >3 times per week); family history of gastric cancer (yes and no); gender (male and female); regular NSAID use for at least 1 year (yes and no); smoking status (no, former, and current); type of gastric polyps (hyperplastic, fundic gland, adenomatous, and others); vegetarian diet (yes and no); waist circumference (<80, 80–90, and >90 cm); and Helicobacter pylori infection (yes, no, and not done). To determine the clinical factors that can independently predict gastric polyp development, the variables identified as significant through univariate analysis were assessed through stepwise logistic regression. A *p* value <0.05 was considered significant, and all analyses were computed on SPSS version 20.0 (SPSS Inc. Chicago, II, USA).

## 3. Results

### 3.1. Participant Demographics and Upper GI Endoscopic Characteristics

**Figure 1:**
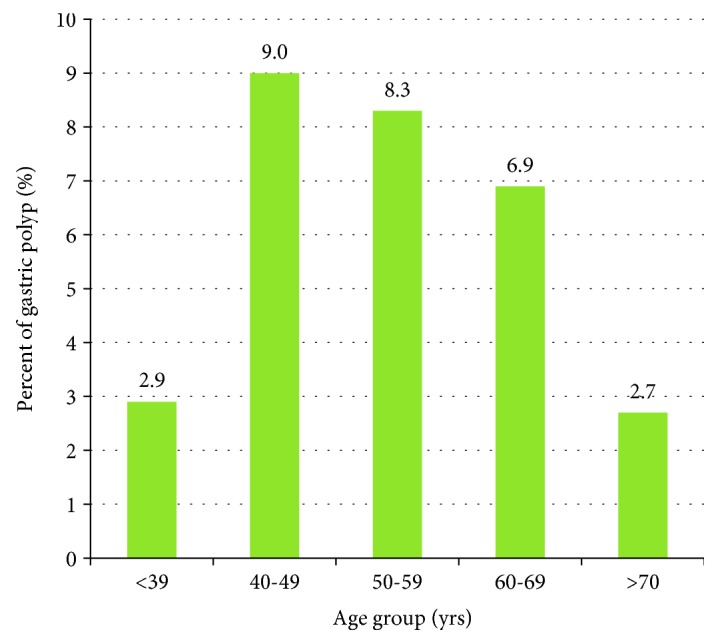
Prevalence of gastric polyps stratified by age group.

In the period October 2015–March 2016, 317 men and 163 women (480 in total) who were asymptomatic participants (of mean age, 52.6 ± 11.3 years; age range, 20–86 years) were enrolled in this study. Of these participants, 143 (29.8%) were diagnosed as having gastric polyps (Table 1). The prevalences of fundic gland, hyperplastic, and adenomatous polyps were 59.4%, 18.2%, and 3.5%, respectively. The pathology of the five adenomatous polyps were all tubular adenoma with low-grade dysplasia. The other types of gastric polyps included 3 carcinoids, 2 inflammatory polys, 2 xanthomas, and others. The prevalences of single polyp and multiple polyps were 53.8% and 46.2%, respectively. The distributions of dimension within the stomach of the polyps were ≤1 cm (97.2%) and >1 cm (2.8%). The prevalence of Helicobacter pylori infection in the biopsied subjects was 19.8%. The age distribution of the participants with gastric polyps is illustrated in Figure 1.

### 3.2. Risk Factors of Gastric Polyp Development

Table 2 summarizes the univariate analysis performed to identify the risk factors of gastric polyps. Age range of 45–60 years, current smoking habit, and lack of regular exercise were significantly associated with the development of gastric polyps (*p* = 0.016, 0.009, and 0.045, respectively). The following data were comparable between participants with and without gastric polyps: BMI; consumption status of alcohol, coffee, spicy food, and tea; consumption status of betel nut; education level; family history of gastric cancer; gender distribution; NSAID use; vegetarian diet; waist circumference; and Helicobacter pylori infection. Age range of 45–60 years (relative risk [RR], 1.59; 95% confidence interval [CI], 1.06–2.40) and current smoking (RR, 1.63; 95% CI, 1.04–2.55) were identified as independent predictors for asymptomatic gastric polyps, as confirmed by the multivariate analysis with stepwise logistic regression (Table 3).

## 4. Discussion

To the best of our knowledge, the present study is the first upper GI endoscopy-based study to investigate the risk factors and prevalence of gastric polyps in asymptomatic Taiwanese. In this study, gastric polyp prevalence in the asymptomatic Taiwanese population was 29.8%, and middle age and current smoking were identified as the relevant risk factors.

Our results differ significantly from those reported in populations in the United States and elsewhere, with the prevalence of gastric polyps being the most salient difference (29.8% in this study vs. 6.35% in the United States [4], 1% in China [8], 0.6% in Brazil [3], and 3–5% in various other countries [12].) These large differences can be ascribed to various intangible and tangible biases, such as differences in the accuracy of the endoscopy as well as its consistency with the pathological diagnosis; in the degree of litigiousness of the studied region; in the demographic, genetic, and socioeconomic characteristics of the study population; and in the study methods.

This finding of the present study provides the latest information about the prevalence of the different polyp types detected in private endoscopy practices in Taiwan and the associations of these polyps with the clinical and demographic characteristics. Fundic gland polyps, which were previously believed to be hamartomatous, accounted for 59.4% of all polyps in this study, which is similar to reports from other countries: 77% in a 2007-2008 nationwide US population-based study [4], 66.1% in a 2010 Chinese study [8], and 47% in a 1994 German study [6]. By contrast, much lower relative prevalence of fundic gland polyps has been reported in other countries, for example, 3.3% in a 1998 Italian study [13] and 16.3% in a 2007 Brazilian study [3]. Individuals with Helicobacter pylori-free stomachs receiving chronic proton-pump inhibitor (PPI) treatment tend to have fundic gland polyps [14–16]. The relative high prevalence of these polyps in our study population may mostly be due to the high PPI use in Taiwan; a likely supporting factor is that endoscopists in Taiwan tend to subject all identified lesions to histopathological analysis. Jalving et al. identified a relationship between long-term PPI (>1 year) therapy and an increased risk of the development of fundic gland polyps; they attributed this relationship to parietal cell hyperplasia as well as protrusions due to acid suppression [17]. In addition, in a retrospective cohort [18], PPI therapy for >48 months was the sole independent predictor of the development of fundic gland polyps; this finding fails to completely account for our findings as PPI therapy for >48 months is rather rare in Taiwan, where 2-3 months is the typical treatment duration that is covered by the National Health Insurance Program, the mandatory national insurance program in Taiwan. Thus, the relationship between PPI use and fundic gland polyp development in Taiwan remains controversial.

Hyperplastic polyps, which have been associated with Helicobacter pylori infection as well as atrophic gastritis [19, 20], represented 18.2% of all polyps in this study. A high (>70%) relative prevalence of hyperplastic polyps was recorded in Greece in 1996 [21] and Brazil in 2007 [3]; a much higher relative prevalence (75%) was found in the United States in 1989 [5], but Carmack et al.'s 2007-2008 study revealed a prevalence of just 17% [4]. Similarly, in China, Cao et al. revealed that hyperplastic polyps, which represented nearly 50% of all polyps in 2000, accounted for just 20.8% in 2010 [8]. This drastic reduction probably due to the fall in the rates of atrophic gastritis and Helicobacter pylori infection.

In this study, current smoking habit was identified as an independent predictor for gastric polyps in asymptomatic participants. Giulio et al. identified that patients with atrophic body gastritis who were also cigarette smokers possessed an odds ratio (OR) of 2.8 (95% CI = 1.2–6.9), relative to nonsmokers, for the development of benign epithelial gastric polyps [19]. Our finding is especially relevant considering that a recent meta-analysis of cohort studies recommended that cigarette smoking be labelled the most influential behavioral risk factor for the development of gastric cancer [22], because cigarette smoke contains numerous human carcinogens (both confirmed and suspected), for example, benzene and polycyclic amines and aromatic amines [23]. This is further substantiated by Martinez et al., who identified APC and KRAS mutations in 36% and 61% of hyperplastic polyps in smokers, respectively, where these mutations were absent in nonsmokers [24]. Moreover, smoking was recently associated with DNA hypermethylation, which in turn has been implicated in the pathogenesis of hyperplastic polyps [25]. In sum, smoking cessation may be a particularly effective form of treatment in such patients.

The following limitations must be considered when applying our findings. Firstly, all health examinations in this study were fully paid for by the participants themselves, an indicator of their higher-than-average economic status relative to the general population of Taiwan; this may have introduced a self-selection bias in our study. Secondly, this study was conducted in a tertiary care facility; thus, the participant may not be representative of patients in primary care facilities. Thirdly, interobserver variability among the pathologists, the subject of numerous investigations in the literature, is a typical source of concern histopathology-based studies. And lastly, detailed information about the description of the localization within the stomach of the polyps and the mucosal status around the polyps, such as mucosal inflammation, atrophy, or intestinal metaplasia, is lacking, which may restrict the outcome.

## 5. Conclusion

This cross-sectional study of asymptomatic individuals revealed a gastric polyp prevalence of 29.8% in Taiwan, with fundic gland polyps accounting for the largest proportion. Middle age and current smoking habit were identified as risk factors of gastric polyp development. Hence, lifestyle modification, such as smoking cessation, may decrease the risk of gastric polyps. Additional studies are necessary to investigate the molecular pathogenesis of gastric polyps and to accordingly determine the appropriate management strategies for the Taiwanese population.

## Figures and Tables

**Table 1:** Demographic data and endoscopic findings of the asymptomatic participants (*n* = 480).

Clinical characteristics	
Age, *n* (%)	
Mean (SD) (years)	52.6 (11.3)
<39	57 (11.9)
40–49	128 (26.7)
50–59	167 (34.8)
60–69	94 (19.6)
>70	34 (7.1)
Height (cm) (SD)	166.3 (8.4)
Weight (kg) (SD)	66.7 (12.7)
Gender, *n* (%)	
Men	317 (66.0)
Women	163 (34.0)
Body mass index, *n* (%)	
Mean (SD)	24.0 (3.2)
<25	314 (65.4)
25–30	147 (30.6)
>30	19 (4.0)
Education level, *n* (%)	
Middle school	53 (11.0)
High school	121 (25.2)
University	239 (49.8)
Graduate school	67 (14.0)
Endoscopic findings, *n* (%)	
Normal	337 (70.2)
Gastric polyps	143 (29.8)
Pathological type	
Fundus	85 (59.4)
Hyperplastic	26 (18.2)
Adenoma	5 (3.5)
Others	27 (18.9)
Numbers	
Single	77 (53.8)
Multiple	66 (46.2)
Size	
≤1 cm	139 (97.2)
>1 cm	4 (2.8)
Helicobacter pylori	
(+)	95 (19.8)
(−)	307 (64.0)
Not done	78 (16.2)

**Table 2:** Univariate analysis of the risk factors of gastric polyp development (*n* = 480).

Principal parameter	G polyps (−)	G polyps (+)	*p* value
Sex, *n* (%)			0.117
Men	230 (68.2)	87 (60.8)	
Women	107 (31.8)	56 (39.2)	
Age (yr.), *n* (%)			0.315
<45	73 (21.7)	37 (25.9)	
>45	264 (78.3)	106 (74.1)	
Age (yr.), *n* (%)			0.016^∗^
Between 45–60	155 (46.0)	81 (57.0)	
Not between 45–60	182 (54.0)	62 (43.0)	
Education (yr.), *n* (%)			0.318
Middle school	33 (9.8)	20 (14.0)	
High school	90 (26.7)	31 (21.7)	
University	164 (48.7)	75 (52.4)	
Graduate school	50 (14.8)	17 (11.9)	
BMI, *n* (%)			0.577
<25	225 (66.8)	89 (62.2)	
25–30	100 (29.7)	47 (32.9)	
>30	12 (3.6)	7 (4.9)	
Waist circumference (%)			0.990
<80 cm	109 (32.3)	45 (31.7)	
80–90 cm	153 (45.4)	65 (45.8)	
>90 cm	75 (22.3)	332 (22.5)	
NSAID use, *n* (%)			0.788
No	324 (96.1)	139 (97.2)	
Yes	13 (3.9)	4 (2.8)	
Family history of gastric cancer, *n* (%)			0.523
No	327 (97.0)	141 (98.6)	
Yes	10 (3.0)	2 (1.4)	
Smoking status, *n* (%)			0.009^∗^
Never smoking	204 (60.5)	68 (47.3)	
Former smoking	53 (15.7)	22 (15.4)	
Current smoking	80 (23.7)	53 (37.3)	
Alcohol drinking, *n* (%)			0.725
No	214 (63.7)	96 (67.1)	
≤3 times per week	99 (29.5)	37 (25.9)	
>3 times per week	23 (6.8)	10 (7.0)	
Coffee drinking, *n* (%)			0.358
No	107 (31.8)	55 (38.5)	
≤3 times per week	73 (21.7)	27 (18.9)	
>3 times per week	157 (46.6)	61 (42.7)	
Tea drinking, *n* (%)			0.688
No	125 (37.1)	56 (39.2)	
≤3 times per week	78 (23.1)	28 (19.6)	
>3 times per week	134 (39.8)	59 (41.3)	
Spicy food consumption, *n* (%)			0.223
No	181 (53.7)	90 (62.9)	
≤3 times per week	94 (27.9)	32 (22.4)	
>3 times per week	62 (18.4)	21 (14.7)	
Betel nut use, *n* (%)			0.186
No	330 (98.2)	141 (99)	
Yes	6 (1.8)	2 (1.0)	
Exercise habit, *n* (%)			0.045^∗^
No	129 (38.3)	68 (47.3)	
≤3 times per week	119 (35.3)	49 (34.2)	
>3 times per week	89 (26.4)	26 (18.5)	
Vegetarian, *n* (%)			0.233
No	319 (97.3)	139 (97.9)	
Yes	9 (2.7)	4 (2.1)	
Helicobacter pylori, *n* (%)			0.218
Not done	53 (15.9)	25 (17.5)	
(−)	215 (63.7)	92 (64.3)	
(+)	69 (23.4)	26 (18.2)	

BMI = body mass index; NSAID = nonsteroidal anti-inflammatory drug. ^∗^*p* < 0.05.

**Table 3:** Independent risk factors of gastric polyp development.

Clinical variable	Coefficient	SE	OR (95% CI^†^)	*p* value
Age 45–60 y/o	0.464	0.209	1.59 (1.06–2.40)	0.027^∗^
Current smoking	0.486	0.229	1.63 (1.04–2.55)	0.034^∗^

^†^CI = confidence interval. ^∗^*p* < 0.05.

## Data Availability

The datasets used and analyzed during the current study are available from the corresponding author on reasonable request.

## Abbreviations

BMI: Body mass index
CI: Confidence interval
DNA: Deoxyribonucleic acid
NSAID: Nonsteroidal anti-inflammatory drug
PPI: Proton-pump inhibitor
RR: Relative risk.
